# Ursolic Acid Suppresses Cholesterol Biosynthesis and Exerts Anti-Cancer Effects in Hepatocellular Carcinoma Cells

**DOI:** 10.3390/ijms20194767

**Published:** 2019-09-26

**Authors:** Geon-Hee Kim, Sang-Yeon Kan, Hyeji Kang, Sujin Lee, Hyun Myung Ko, Ji Hyung Kim, Ji-Hong Lim

**Affiliations:** 1 Department of Applied Life Science, Graduate School of Konkuk University, College of Biomedical & Health Science, Konkuk University, Chungju 27478, Korea; rlarjsgml4@kku.ac.kr (G.-H.K.); hsb6477@kku.ac.kr (S.-Y.K.); kkang@kku.ac.kr (H.K.); 201341532@kku.ac.kr (S.L.); 2 Diabetes and Bio-Research Center, Konkuk University, Chungju 27478, Korea; 3 Department of Life Science, College of Science and Technology, Woosuk University, 66 Daehak-ro, Jincheon-eup, Chungcheongbuk-do 27841, Korea; greatmen00@hanmail.net; 4 College of Life Sciences and Biotechnology, Korea University, Seoul 02841, Korea; jay_kim@korea.ac.kr

**Keywords:** Ursolic acid, SREBP2, Cholesterol, Hepatocellular carcinoma

## Abstract

Abnormally upregulated cholesterol and lipid metabolism, observed commonly in multiple cancer types, contributes to cancer development and progression through the activation of oncogenic growth signaling pathways. Although accumulating evidence has shown the preventive and therapeutic benefits of cholesterol-lowering drugs for cancer management, the development of cholesterol-lowering drugs is needed for treatment of cancer as well as metabolism-related chronic diseases. Ursolic acid (UA), a natural pentacyclic terpenoid, suppresses cancer growth and metastasis, but the precise underlying molecular mechanism for its anti-cancer effects is poorly understood. Here, using sterol regulatory element (SRE)-luciferase assay-based screening on a library of 502 natural compounds, this study found that UA activates sterol regulatory element-binding protein 2 (SREBP2). The expression of cholesterol biosynthesis-related genes and enzymes increased in UA-treated hepatocellular carcinoma (HCC) cells. The UA increased cell cycle arrest and apoptotic death in HCC cells and reduced the activation of oncogenic growth signaling factors, all of which was significantly reversed by cholesterol supplementation. As cholesterol supplementation successfully reversed UA-induced attenuation of growth in HCC cells, it indicated that UA suppresses HCC cells growth through its cholesterol-lowering effect. Overall, these results suggested that UA is a promising cholesterol-lowering nutraceutical for the prevention and treatment of patients with HCC and cholesterol-related chronic diseases.

## 1. Introduction

Hepatocellular carcinoma (HCC) is the most common type of primary liver cancer, which is closely associated with chronic liver diseases, particularly viral hepatitis and metabolic steatohepatitis, and is the third most common cause of cancer-related deaths worldwide [1,2,3].

Ursolic acid (UA) is a pentacyclic terpenoid and a secondary plant metabolite, usually found in the holy basil (*Ocimum sanctum* L.), thyme (*Thymus vulgaris* L.), lavender (*Lavandula augustifolia*), catnip (*Nepeta sibthorpii*), peppermint leaves (*Mentha piperita* L.), or fruit peel [4,5]. It has various benefits for the prevention and treatment of chronic human diseases, such as diabetes, cardiovascular, arthritis, atherosclerosis, obesity, and cancer [5].

UA is known to induce cell cycle arrest and apoptosis, suppress angiogenesis and metastasis, and diminish chemoresistance in several cancers, including lung cancer [6,7], breast cancer [8,9], prostate cancer [10], colon cancer [11,12], liver cancer [13,14], gastric cancer [15], and melanoma [16,17]. In addition, the anti-cancer effects of UA have been observed in animal models, such as subcutaneous xenograft (HCT116 and A549), orthotopic xenograft (HCT116 and Panc-28), transgenic adenocarcinoma of mouse prostate (TRAMP), and DMBA-induced skin cancer [12,17,18,19].

The evidences from previous studies suggest that UA exerts its anti-cancer effects through the suppression of oncogenic growth signaling, such as that via phosphoinositide 3-kinase (PI3K)/protein kinase B (AKT) and epidermal growth factor receptor (EGFR)/mitogen-activated protein kinase (MAPK) pathways, and oncogenic transcription factors, such as nuclear factor kappa-light-chain-enhancer of activated B cells (NF-kB), signal transducer and activator of transcription 3 (STAT3), and hypoxia-inducible factor-1α (HIF-1α), in several types of cancer [5]. However, the precise molecular mechanism by which UA affects these cancer-promoting signaling molecules and transcription factors is poorly understood.

Mammalian cells synthesize cholesterol through a series of 21 enzymatic steps, including the mevalonate (MVA) pathway, generating various metabolites that are required for maintenance of physiological and developmental processes [20]. Enriched cholesterol is commonly observed in lipid raft microdomains of the cell membrane, which is involved in various cellular functions, such as the regulation of cell adhesion, migration, and growth signaling, e.g., PI3K/AKT and EGFR/MAPK [21,22,23]. Therefore, the increase in intracellular cholesterol level due to dysregulation of its biosynthetic pathways is a common feature of cancer, and the evidence suggests that cholesterol is a critical component in the progression of various cancers, including breast, prostate, liver, and colorectal cancer [21,24].

Although the main source of cholesterol is diet, intracellular cholesterol levels are carefully regulated and balanced by sterol regulatory element-binding protein 2 (SREBP2)-mediated transcriptional programming [25]. When intracellular cholesterol levels are sufficient, SREBP2 is not processed to its maturation, and cholesterol synthesis is not stimulated. Conversely, when the cells sense low cholesterol levels, SREBP2 maturation is induced, followed by its translocation into the nucleus for activation of its target genes, including those encoding hydroxymethylglutaryl (HMG)-CoA synthase 1 (HMGCS1), HMG-CoA reductase (HMGCR), farnesyl diphosphate synthase (FDPS), and mevalonate diphosphate decarboxylase (MVD), for de novo cholesterol synthesis [25].

Statins, inhibitors of HMG-CoA reductase, which is the rate-limiting enzyme in cholesterol biosynthesis, are widely used as cholesterol-lowering drugs [26,27]. Emerging evidence from tissue culture, animal, and clinical studies indicates that several statins, such as strovastatin, fluvastatin, and simvastatin, stimulate cell cycle arrest, apoptotic cell death, and the suppression of EMT and cancer stemness in several types of proliferating cancer cells, including hepatocellular carcinoma, breast, prostate, glioma, ovarian, and colorectal cancer cells [28,29,30,31]. Recent meta-analyses have revealed that statins have a beneficial effect with respect to reduced cancer-related mortality on multiple cancer types, including hepatocellular carcinoma (HCC), breast, lung, prostate, colorectal, and kidney cancer [32,33,34,35,36,37,38,39]. Manthravadi et al. reported that statin use is associated with improved recurrence-free survival (RFS), cancer-specific survival, and overall survival in breast cancer patients [33]. A meta-analysis in patients with HCC in a U.S. population revealed that ever-use of statins has a significant inverse association with HCC development [34]. In addition, a reduced cancer-related mortality among statin users, in comparison with those who had never used statins, was observed for 13 cancer types [40]. Consistent with these observations, accumulated data from a meta-analysis revealed that statins may have beneficial effects for the prevention and treatment of multiple types of cancer.

A growing body of preclinical, epidemiological, and clinical evidence has shown the various side effects of the most commonly used cholesterol-lowering nutraceuticals [41]. However, efforts to develop cholesterol-lowering nutraceuticals, which exert anti-cancer effects without side effects, are needed for effective cancer prevention and treatment. The present study identified UA as a cholesterol-lowering drug using a library of 502 natural compounds. In addition, transcriptional activation of SREBP2 and its cholesterol biosynthesis-related target genes in UA-treated HCC cells was observed. Consistent with this cholesterol-lowering effect of UA, this study also found that UA had a negative effect on the oncogenic AKT and MAPK signaling pathway and cell viability, thereby increasing the incidence of cell cycle arrest and apoptotic death in HCC cells. Overall, our results suggest that UA may be a promising cholesterol-lowering drug to achieve prevention and treatment in patients with HCC.

## 2. Results and Discussion

### 2.1. UA Identified as A SREBP2 Activator

**Figure 1:**
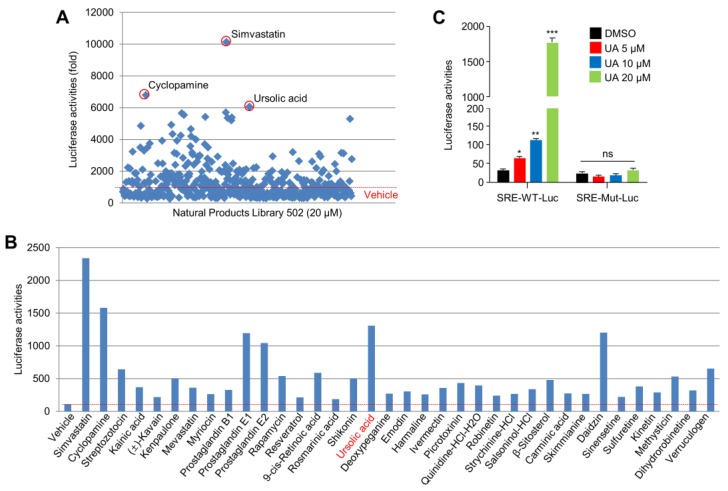
Luciferase assay-based screening for natural compounds that activate the sterol regulatory element-binding protein 2 (SREBP2). (**A**) Wild-type sterol regulatory element (SRE)-luciferase vector was transiently expressed in HEK293T cells, and incubated for 24 h with dimethyl sulfoxide (DMSO), as a negative control, or one of the 502 natural compounds. Each compound was dissolved in dimethyl sulfoxide (DMSO) and used at a final concentration of 20 μM. The values represent the mean from the two independent experiments performed. (**B**) HEK293T cells expressing SRE-luciferase vector were incubated for 24 h with 25 of the 502 library compounds, selected during the first screening. Simvastatin (20 μM) was used as a positive control and DMSO (vehicle) was used as a negative control. The values represent the mean from two independent experiments performed in triplicate. (**C**) SK-HEP-1 cells expressing SRE-WT (wild type of SRE) or SRE-MT (mutant of SRE)-luciferase vector were incubated for 24 h with ursolic acid (UA) at different concentrations, as indicated. The values represent the mean ± SD of three independent experiments performed in triplicate; * *p* < 0.05, ** *p* < 0.01, and *** *p* < 0.001.

Sterol regulatory element-binding protein 2 (SREBP2) undergoes maturation and activation upon cholesterol depletion, subsequently triggering the enhancement of intracellular cholesterol biosynthesis, for maintaining cholesterol homeostasis [25]. Paradoxically, statins, cholesterol-lowering drugs, activate SREBP2 and its target genes related to cholesterol biosynthesis, such as HMGCS1, HMGCR, MVD, and FDPS, in order to increase intracellular cholesterol levels [42,43]. Thus, screening for a transcriptional activator of SREBP2 is a promising way to identify novel cholesterol-lowering small molecules. Here, this study attempted to identify a potential SREBP2 transcriptional activator using a SREBP2-binding sequence containing a sterol-responsive element (SRE), by conducting a luciferase assay on a library of 502 natural compounds. This study identified 24 compounds that increased the SRE luciferase activity to levels higher than that of the vehicle, which served as a negative control (Figure 1A). An additional luciferase assay to validate the larger screening also showed that these 24 compounds activated SRE-luciferase activities (Figure 1B). In this screening, UA, daidzin, and prostaglandins were found to highly activate the SRE-luciferase activity. In addition, a dose-dependent increase in SRE-luciferase activity was also observed in UA-treated SK-HEP-1, hepatocellular carcinoma (HCC) cells (Figure 1C), suggesting that UA is a potential cholesterol-lowering and SREBP2-activating natural compound. Consistent with our finding, the evidence from multiple sources suggests that UA, as a pentacyclic terpenoid, exhibits various beneficial pharmacological effects against metabolism-related chronic diseases, such as obesity [44], liver failure [45], and skeletal muscle atrophy [46]. Indeed, previous reports have shown that UA significantly decreases the blood cholesterol and triglyceride levels in a high-fat-diet-induced obesity animal model [47,48]. UA has also been found to decrease the liver triglyceride and free fatty acid levels in hepatic steatosis and liver fibrosis models [49,50]. Thus, the identification of UA as a SREBP2 activating small molecule could provide useful insights into the precise molecular mechanism underlying the beneficial effects of UA in various chronic human diseases.

### 2.2. UA Induces the Expression of Cholesterol Biosynthesis-Related Genes and Enzymes

**Figure 2:**
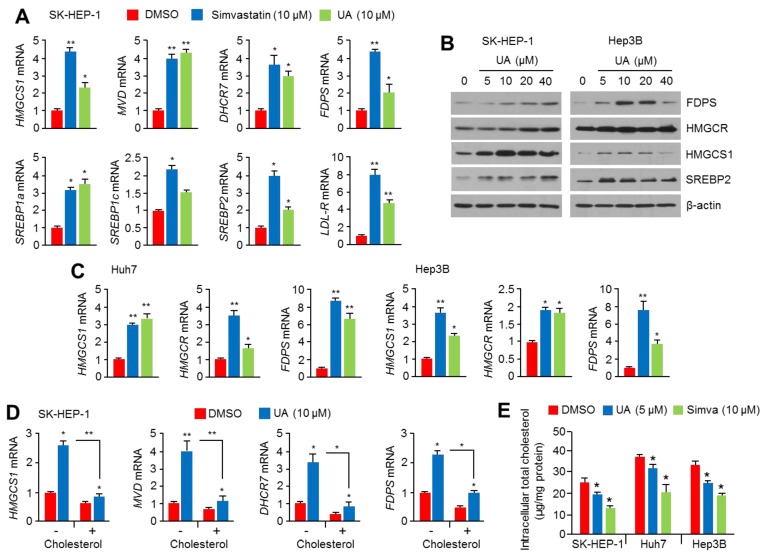
UA increases the expression of cholesterol biosynthesis-related genes and enzymes in hepatocellular carcinoma (HCC) cells. (**A**) SK-HEP-1 cells were incubated with UA (20 μM) or simvastatin (10 μM) for 24 h. The mRNA levels were measured by quantitative real-time polymerase chain reaction (PCR). The values represent the mean ± SD from three independent experiments performed in duplicate; * *p* < 0.05 and ** *p* < 0.01. (**B**) SK-HEP-1 and Hep3B cells were incubated with UA for 24 h at different concentrations, as indicated. Protein expression was measured by western blotting. (**C**) Huh7 and Hep3B cells were incubated with UA (20 μM) or simvastatin (10 μM) for 24 h, following which, the mRNA levels were measured. The values represent the mean ± SD from three independent experiments performed in duplicate; * *p* < 0.05 and ** *p* < 0.01. (**D**) SK-HEP-1 cells were incubated with or without 0.5 mM of water-soluble cholesterol for 6 h prior to UA (20 μM) treatment, followed by further incubation with DMSO or UA (20 μM) for 24 h. The values represent the mean ± SD from two independent experiments performed in triplicate; * *p* < 0.05 and ** *p* < 0.01. (**E**) Intracellular total cholesterol levels were measured in UA or simvastatin-treated SK-HEP-1, Huh7 and Hep3B cells. UA or simvastatin was treated for 48 h prior to measure intracellular total cholesterol. The values are represented as the mean ± SD of three independent experiments performed in duplicate; * *p* < 0.05.

To investigate whether UA activates SREBP2, the expression of cholesterol biosynthesis-related genes was analyzed in the absence or presence of UA. The increased expression was observed for genes related to cholesterol biosynthesis (HMGCS1, HMGCR, MVD, FDPS, and SREBP2), fatty acid synthesis (SREBP1a and SREBP1c), and cholesterol uptake (LDL-R) in UA-treated SK-HEP-1 cells, and this UA-induced gene expression was similar to that observed with simvastatin treatment (Figure 2A). Given that increased expression of SREBP2 and cholesterol biosynthesis-related genes is a compensatory mechanism induced by cholesterol-lowering drugs [51,52], our results indicate that UA also had cholesterol-lowering effects similar to statins. Similarly, the increased expression of proteins such as FDPS, HMGCR, HMGCS1, and SREBP2 was observed in UA-treated SK-HEP1 and Hep3B cells (Figure 2B). UA was also found to increase the expression of cholesterol biosynthesis-related genes in Huh7 and Hep3B cells (Figure 2C), suggesting that the activation of SREBP2 and the expression of its target genes upon UA treatment may be a general effect, at least in HCC cells. Therefore, the next crucial question is whether the observed increase in the expression of cholesterol biosynthesis-related genes is indeed caused by the cholesterol-lowering effect of UA. To address this question, the expression of cholesterol biosynthesis-related genes upregulated by UA treatment was analyzed in the absence or presence of water-soluble cholesterol supplementation. Interestingly, the supplementation of water-soluble cholesterol was found to diminish the upregulation of cholesterol biosynthesis-related genes by UA treatment (Figure 2D). In addition, this study examined whether UA can act like statins to decrease intracellular cholesterol level. Interestingly, the decreased intracellular cholesterol levels were observed in 5 μM of UA-treated SK-HEP-1, Huh7 and Hep3B cells (Figure 2E). These results indicate that UA increases the expression of cholesterol biosynthesis-related genes, and this may be caused by its cholesterol-lowering effect in HCC cells.

### 2.3. UA Attenuates Growth-Signaling Pathways in A Cholesterol-Dependent Manner

**Figure 3:**
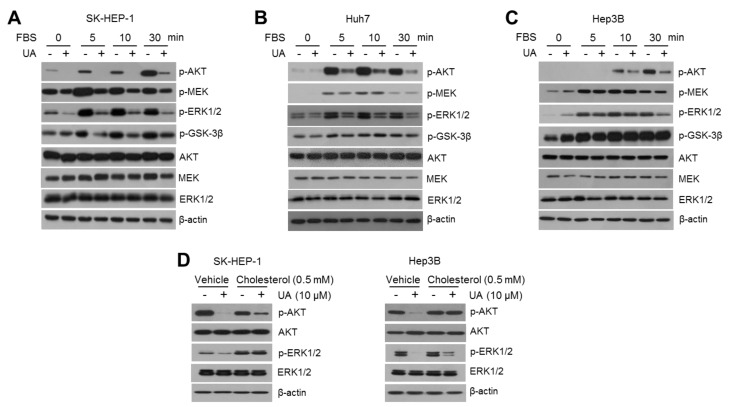
Ursolic acid (UA) increases the expression of cholesterol biosynthesis-related genes and enzymes in hepatocellular carcinoma (HCC) cells. (**A**) SK-HEP-1 cells were incubated, upon fetal bovine serum (FBS) starvation, for 24 h prior to UA treatment. After serum starvation, the cells incubated with DMSO or UA (20 μM) in the absence or presence of 10% FBS, as indicated. The phosphorylation of growth signaling pathway-related proteins were measured by western blotting. (**B**) Suppressive effect of UA on growth signaling measured in Huh7. (**C**) Suppressive effect of UA on growth signaling measured in Hep3B. (**D**) SK-HEP-1 and Hep3B cells were incubated for 2 h with or without 0.5 mM of water-soluble cholesterol prior to UA treatment, and cells were then further incubated with DMSO or UA (20 μM) for 24 h. Phosphorylation of AKT and ERK1/2 was measured using western blotting.

Cholesterol, a component of lipid rafts, promotes cell growth signaling factors, such as PI3K/AKT and EGFR/MAPK, by mediating the trafficking of oncogenic growth factor receptors [24]. In addition, multiple types of metabolites and intermediates generated from mevalonate pathway and cholesterol biosynthesis are critical for the membrane anchoring of oncogenic signaling proteins such as RAS, PI3K, and AKT [53]. Therefore, this study investigated whether UA could attenuate growth-factor-mediated oncogenic growth signaling pathways. As expected, the starvation of fetal bovine serum (FBS), which contains various growth factors, sufficiently decreased the phosphorylation of AKT, MEK, and ERK1/2, thereby increasing the FBS supplementation dramatically in these signaling pathways (Figure 3A–C). Interestingly, UA was found to suppress the phosphorylation of AKT, MEK, and ERK1/2 by growth factors-enriched FBS in SK-HEP-1 (Figure 3A), Huh7 (Figure 3B), and Hep3B (Figure 3C) cells. As hyperactivation of oncogenic signaling caused by gain-of-function genetic mutations such as PI3K, RAS, MYC, and RAF, is a hallmark of cancer development and progression [54], these results suggest that the suppressive effect of UA on oncogenic growth signaling is a critical insight for the development of UA-based anti-cancer therapeutics. However, there remains a question as to how UA downregulates the growth-factor-induced oncogenic signaling pathways. To understand this question, this study investigated whether cholesterol is responsible for the suppression of oncogenic growth signaling caused by UA. Figure 3D shows that the supplementation with water-soluble cholesterol was sufficient to reverse UA-induced attenuation of growth signaling in SK-HEP-1 and Hep3B cells. These results indicate that the cholesterol-lowering effect of UA is a possible regulatory mechanism by which UA suppresses oncogenic growth signaling in HCC cells.

### 2.4. UA Decreases Viability of Hepatocellular Carcinoma (HCC) Cells

**Figure 4:**
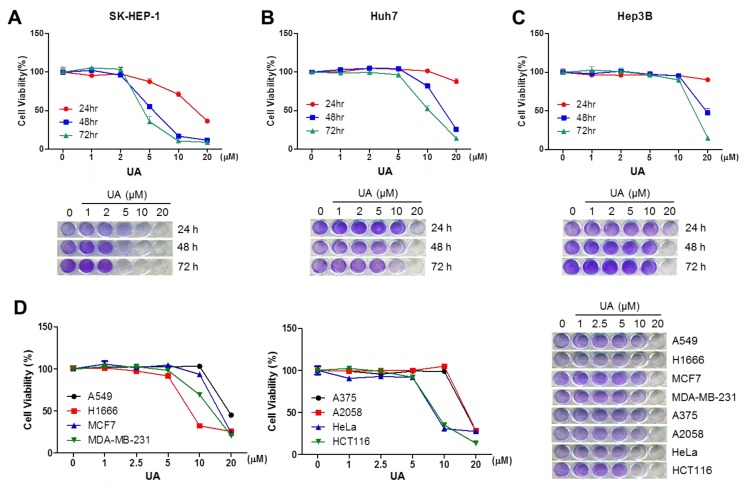
UA attenuates cell viability in HCC cells. (**A**) SK-HEP-1 cells were incubated with UA at different concentrations for 24, 48, and 73 h, as indicated. (**B**) Attenuation of cell viability by UA treatment was measured in Huh7 cells. (**C**) Attenuation of cell viability by UA treatment was measured in Hep3B cells. (**D**) Attenuation of cell viability by UA treatment was measured in lung (A549 and H1666), breast (MCF7 and MDA-MB-231), melanoma (A375 and A2058), cervical (HeLa) and colon (HCT116) cancer cells. Cell viability was measured by crystal violet staining, and the values represent the mean from three independent experiments performed in duplicate.

As UA dramatically suppresses the oncogenic growth signaling, the growth-suppressive effect of UA in HCC cells was further tested. Here, this study found that UA, at a concentration of 10–20 μM, strongly inhibits cell viability in SK-HEP-1 (Figure 4A), Huh7 (Figure 4B), and Hep3B (Figure 4C) cells. Although the regulatory mechanism by which UA exerts anti-proliferative effects is not clear, large numbers of previous evidences have also shown that UA attenuates cell growth in multiple types of cancer cells, such as colorectal [11], breast [55], gastric [56], and melanoma [57]. Consistently, our results also show that UA was sufficient to decrease cell viability in lung (A549 and H1666), breast (MCF7 and MDA-MB-231), melanoma (A375 and A2058) and cervical (HeLa) cancer cells (Figure 4D). Thus, our findings, as well as those from other studies, support the potential efficacy of UA as a natural compound for the prevention and treatment of HCC. Previous reports have shown that 20 mg/kg of UA exerts anti-cancer effects in TC-1 (cervical cancer cells) and SW620 (colorectal cancer cells) transplanted mice models [58,59]. These backgrounds encourage the investigation of whether 20 mg/kg of UA effectively suppresses the growth of HCC and liver neoplasm by inhibiting mevalonate and cholesterol biosynthesis pathways in vivo models.

### 2.5. UA Promotes Cell Cycle Arrest and Apoptotic Death in HCC Cells

**Figure 5:**
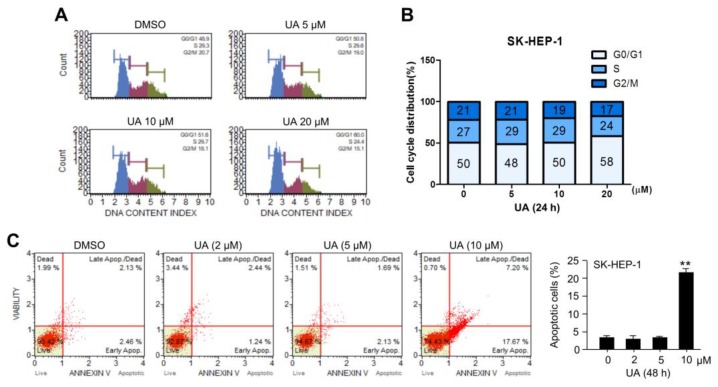
UA causes cell cycle arrest and apoptotic death in HCC cells. (**A**,**B**) SK-HEP-1 cells were incubated for 12 h with DMSO or UA at different concentrations, as indicated. Cell cycle was assessed using the Muse™ cell cycle assay kit. The values represent the mean from three independent experiments performed in duplicate. (**C**) SK-HEP-1 cells were incubated for 48 h with DMSO or UA at different concentrations, as indicated. Apoptotic cell death was measured using Muse™ Annexin V and Dead Cell kits. The values represent the mean ± SD from three independent experiments performed in duplicate; ** *p* < 0.01.

The insufficient cholesterol biosynthesis and supplementation increases the incidence of cell cycle arrest and apoptotic cell death [60,61]. In addition, cholesterol-lowering drugs, such as simvastatin and lovastatin, have also been found to promote cell cycle arrest and apoptosis in various cancer cells [62,63,64,65]. Here, whether UA acts similarly to cholesterol-lowering drugs to promote cell cycle arrest and apoptotic cell death in HCC cells was investigated. Figure 5A,B shows that UA promotes cell cycle arrest at G0/G1 checkpoint in a dose-dependent manner. Consistent with this, approximately 20% increase in apoptotic cells upon treatment with 20 μM UA, was observed in SK-HEP-1 cells (Figure 5C). These results indicate that UA causes cell cycle arrest and apoptotic cell death in HCC cells.

### 2.6. Anti-Cancer Effect of UA in HCC Cells is Diminished by Cholesterol Supplementation

**Figure 6:**
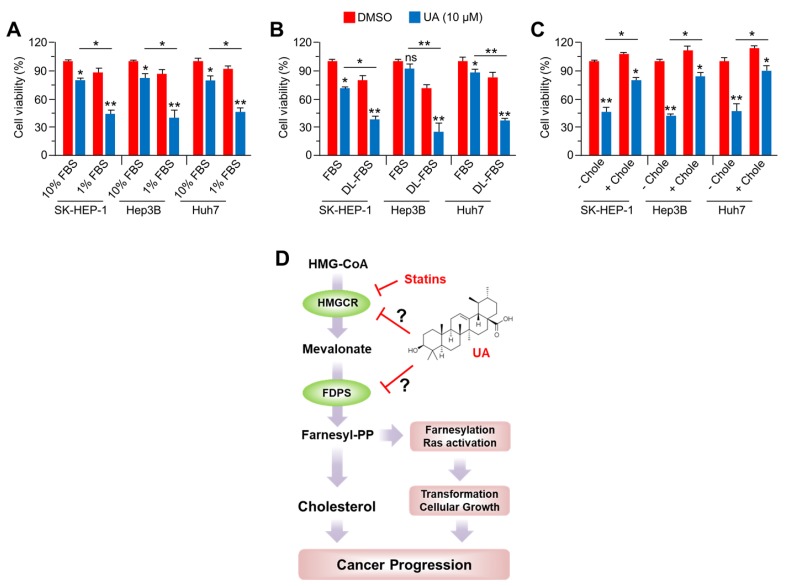
Cholesterol is required for the anti-cancer effect of UA. (**A**) Three cell lines (SK-HEP-1, Hep3B, and Huh7) were incubated with DMSO or UA (20 μM) for 24 h with 10% FBS or 1% FBS. Cell viability was measured by crystal violet staining, and the values represent the mean ± SD from three independent experiments performed in triplicate; * *p* < 0.05 and ** *p* < 0.01. (**B**) Cells were incubated with DMSO or UA (20 μM) for 24 h with 10% normal FBS or lipoprotein-depleted FBS (DL-FBS). The values represented the mean ± SD from three independent experiments performed in triplicate; * *p* < 0.05 and ** *p* < 0.01. (**C**) Cells were incubated with or without 0.5 mM water-soluble cholesterol for 2 h prior to UA (20 μM) treatment, followed by further incubation with DMSO or UA (20 μM) for 24 h with 1% FBS. The values represent the mean ± SD from two independent experiments performed in triplicate; * *p* < 0.05 and ** *p* < 0.01. (**D**) Speculated molecular mechanism of anti-cancer effects of UA.

To understand whether UA suppresses HCC cell growth through a cholesterol-lowering effect, the alteration in cell growth by UA was further measured in the absence or presence of cholesterol. Initially, a strong suppression of cell viability by UA upon FBS deprivation, was observed in SK-HEP-1, Hep3B, and Huh7 cells. This indicates that FBS, containing high levels of cholesterol in the form of lipoproteins may modulate the anti-cancer effects of UA (Figure 6A). Importantly, Figure 6B shows that UA attenuated the cell growth more effectively in the presence of lipoprotein-depleted FBS (DL-FBS) than in the culture medium containing normal FBS. As direct evidence of the fact that the cholesterol-lowering effect is a critical mechanism for the anti-cancer effect of UA, a decreased cell viability by UA was confirmed upon additional cholesterol supplementation. Consistent with Figure 6A,B, water-soluble cholesterol supplementation significantly reverses UA-induced attenuation of cell growth in HCC cells (Figure 6C). The evidence from previous studies indicates that UA inhibits cancer growth through its effects on oncogenic growth signaling and several cancer-promoting transcription factors, such as STAT3, NF-Kb, and HIF-1α [5]. These results suggest that UA suppresses the oncogenic growth signaling through cholesterol-lowering effects, resulting in an attenuated growth of HCC cells. However, the critical regulatory mechanism by which UA attenuates cancer growth is poorly understood. Here, the authors speculate several possible regulatory mechanisms (Figure 6D). First, UA acts as an inhibitor of HMGCR similar to those of statins. Indeed, our results showed that UA decreased intracellular cholesterol levels, but paradoxically increased mevalonate and cholesterol biosynthesis-related genes expression, which are commonly observed in statins-treated cells. Second, UA could suppress FDPS-mediated production of FPP and GGPP, which activate oncogenic growth signaling and cellular transformation through farnesylation of oncogenic proteins including Ras [24]. In fact, it was found that oncogenic growth signaling such as RAS-MAPK and PI3K-AKT signaling pathways was diminished by UA treatment. Thus, these speculated regulatory mechanisms by which UA acts as an inhibitor against HMGCR and FDPS needed further in-depth investigations.

## 3. Materials and Methods

### 3.1. Reagents and Antibodies

A library of 502 natural compounds was obtained from Enzo Biochem (Farmingdale, NY, USA). Ursolic acid (U6753), water-soluble cholesterol (C4951) and simvastatin (S6196) were purchased from Sigma Aldrich (St. Louis, MO, USA) and Santa Cruz Biotechnology (Dallas, TX, USA). The antibodies recognizing HMGCS1 (CST-36877), HMGCR (ab174830), FDPS (ab189874), SREBP2 (ab30682), AKT (CST-4691), phospho-AKT (CST-4060), MEK (CST-9122), phospho-MEK (CST-9154), ERK1/2 (sc-94), phospho-ERK1/2 (CST-4370), phospho-GSK3β (CST-5558) and β-actin (sc-47778) were purchased from Cell Signaling Technology (Danvers, MA, USA), Santa Cruz Biotechnology, and Abcam (Cambridge, MA, USA).

### 3.2. Cell Culture and Cell Viability Assay

Hepatocellular carcinoma (Hep3B, SK-HEP-1 and Huh7), lung adenocarcinoma (A549 and NCI-H1666), malignant melanoma (A375 and A2058), breast cancer (MCF7 and MDA-MB-231) and cervical cancer (HeLa) cells were obtained from the Korean Cell Line Bank (Seoul, Korea) and American Type Culture Collection (Manassas, VA, USA) and cultured in Dulbecco’s modified Eagle’s medium (DMEM) and Minimum Essential Medium Eagle alpha medium (MEM-α) supplemented with 10% fetal bovine serum (FBS) and antibiotics. Lipoprotein depleted FBS (d ≤ 1.25 g/mL), which produced and qualified by ultracentrifugation and agarose gel electrophoresis, respectively, (DL-FBS, Kalen Biomedical, Germantown, MD, USA) was used for cholesterol deprivation. Cell viability was measured as previously described [1]. To measure cell viability, the Hep3B, SK-HEP-1 and Huh7 cells were seeded into 24-well tissue culture dishes and incubated for 24, 48, and 72 h with or without ursolic acid (UA) upon 10% FBS, 1% FBS, lipoprotein depletion and/or supplementation of water-soluble cholesterol. The cultured cells were washed and fixed with phosphate-buffered saline (PBS) and 4% paraformaldehyde. The fixed cells were incubated with crystal violet solution for 20 min at room temperature, and the stained cells were solubilized in 1% SDS solution. The optical density was measured at 570 nm by using an absorbance reader (BioTek, Winooski, VT, USA) (OD570).

### 3.3. Western Blotting

For western blotting, total protein samples were extracted by using a protein extraction buffer (1% IGEPAL, 150 mM NaCl, 50 mM Tris-HCl (pH 7.9), 10 mM NaF, 0.1 mM EDTA, and a protease inhibitor cocktail) as previously described [1]. The total proteins were separated followed by molecular weight by using sodium dodecyl sulfate (SDS)-polyacrylamide gel electrophoresis (PAGE). The separated proteins were then transferred onto PVDF membranes (Millipore, Burlington, MA, USA), and the transferred membranes reacted with primary antibodies (1:1,000–1:5,000) and horseradish peroxidase (HRP)-conjugated secondary antibodies (1:10,000) at 4 °C and room temperature for 12 h and 1 h, respectively. The Enhanced Chemiluminescence (ECL) Prime kit (GE Healthcare, Pittsburgh, PA, USA) was used for visualization of the differences of protein expression.

### 3.4. Quantitative Real-Time PCR

The mRNA encoding cholesterol biosynthesis-related enzymes expression was measured by quantitative real-time polymerase chain reaction (PCR) performed as previously described [1]. Total RNA was extracted by using TRIzol (Invitrogen, Carlsbad, CA, USA). The cDNA was synthesized by using a high-capacity cDNA reverse transcription kit (Applied Biosystems, Waltham, MA, USA). Quantitative PCR was performed by using SYBR Green PCR Master Mix (Applied Biosystems, Waltham, MA, USA). Human 36B4 (rplp0, acidic ribosomal phosphoprotein P0) gene was used for housekeeping control. The primer sequences used in the experiment are shown in Table 1.

### 3.5. Cell Cycle Analysis

The cell cycle analysis was performed as previously described [1]. The cultured cells in the absence or presence of UA were harvested and fixed using cold PBS and 70% ethanol, and the cells were then incubated for 3 h at −20 °C. The fixed cells were incubated with Muse™ cell cycle assay kit reagent (200 µL) for 30 min at room temperature. The cell populations were analyzed by using a Mini Flow Cytometry Muse™ Cell Analyzer (Millipore, Burlington, MA, USA).

### 3.6. Apoptosis Assays

Annexin-V staining to measure apoptotic cell death was performed as previously described [1]. The culture cells (1 × 10^5^ cells/well) in the absence or presence of UA were collected into fresh tubes, and the cells were then washed with cold PBS and centrifuged at 2000 rpm for 2 min at room temperature. The cell pellets were mixed and reacted with 100 μL of Muse™ Annexin V and Dead Cell kit reagents (Millipore, Burlington, MA, USA) for 20 min. The apoptotic cells population was measured by using Mini Flow Cytometry Muse™ Cell Analyzer (Millipore, Burlington, MA, USA).

### 3.7. Luciferase Assay

Luciferase assay was performed as previously described [1]. HEK293T cells transiently transfected with steroid responsive element (SRE)-wild type (WT) or -mutant (Mut) containing luciferase vector (pSynSRE-T-Luc and pSynSRE-Mut-T-Luc) by using Lipofectamine 2000 (Invitrogen, Carlsbad, CA, USA). pSynSRE-T-Luciferase vectors (Addgene plasmid #60444 and #60490) were gifts from Timothy Osborne [66]. To screen the SREBP2 activating natural compounds, transfected HEK293T cells were incubated with a library of 502 natural compounds (20 μM of each compounds) for 24 h. Luciferase activities were analyzed by using a Synergy 2 Luminometer (BioTek, Winooski, VT, USA) and β-gal assay was used for normalization.

### 3.8. Measurement of Intracellular Cholesterol

Intracellular total cholesterol levels were measured by using the Amplex Red cholesterol assay kit (Invitrogen, Carlsbad, CA, USA) in accordance with the manufacturer’s protocols as previously described [1]. Briefly, UA or simvastatin-treated SK-HEP-1, Huh7 and Hep3B cells were harvested and kept at −70 °C. The cells were lysed with reaction buffer and sonication to disrupt the cellular membrane. The cell lysates (50 µL) were mixed and reacted for 30 min at 37 °C with horseradish peroxidase (HRP), cholesterol oxidase and cholesterol esterase containing Amplex Red reagent, and then the fluorescence intensity was measured by using a fluorescence microplate reader (BioTek, Winooski, VT, USA) at ex/em = 530/590.

### 3.9. Statistical Analysis

The data are represented as the mean ± standard deviation (SD). All statistical analyses were performed using two-tailed Student’s *t*-test and one-way ANOVA with Tukey post hoc test. A *p* value of < 0.05 was considered statistically significant.

## 4. Conclusions

Despite the accumulating evidence that UA exerts an anti-cancer effect in multiple types of cancer, the precise underlying molecular mechanism is not clear. The major finding of this study is that UA exerts its anti-cancer effect through a cholesterol-lowering effect in hepatocellular carcinoma (HCC) cells. Taken together, these results indicate that UA, as a promising cholesterol-lowering drug, may be useful for the development of drugs and/or functional foods for the prevention and treatment of HCC as well as dysregulated cholesterol metabolism-related chronic diseases.

## Figures and Tables

**Table 1:** Primer sequences for quantitative real time-PCR.

Gene	Forward Primer	Reverse Primer
**HMGCS1**	TGGCAGGGAGTCTTGGTA	TCCCACTCCAAATGATGACA
**HMGCR**	GATGGGAGGCCACAAAGAG	TTCGGTGGCCTCTAGTGAGA
**MVD**	TTAACTGGTCCTGGTGCAGA	AACATCGCGGTCATCAAGTA
**FDPS**	TCCATGATGTCATCTGCCAC	AGCCAAGGAAACAGGATG
**SREBP1a**	GCACCCACTCCATTGAAGAT	GGCACTGACTCTTCCTTGATAC
**SREBP1c**	ACAGTGACTTCCCTGGCCTAT	GCATGGACGGGTACATCTTCA
**SREBP2**	AACGGTCATTCACCCAGGTC	GGCTGAAGAATAGGAGTTGCC
**LDL-R**	AACTGCCATTGTCGTCTTTA	ACATACCCATCAACGACAAG
**36B4**	CATGTTGCTGGCCAATAAGG	TGGTGATACCTAAAGCCTGGAA
